# Biocatalytic synthesis and ordered self-assembly of silica nanoparticles via a silica-binding peptide

**DOI:** 10.3762/bjnano.14.25

**Published:** 2023-02-28

**Authors:** Mustafa Gungormus

**Affiliations:** 1 Biomedical Engineering, School of Engineering and Natural Sciences, Ankara Yildirim Beyazit University, Ankara, Türkiye; 2 MERLAB Application and Research Center, Ankara Yildirim Beyazit University, Ankara, Türkiye

**Keywords:** biocatalysis, biomimetics, nanoparticle, peptide, self-assembly, silica

## Abstract

Achieving scalable and economic methods for manufacturing ordered structures of nanoparticles is an ongoing challenge. Ordered structures of SiO_2_ nanoparticles have gained increased attention due to the great potential they offer in filtering, separation, drug delivery, optics, electronics, and catalysis. Biomolecules, such as peptides and proteins, have been demonstrated to be useful in the synthesis and self-assembly of inorganic nanostructures. Herein, we describe a simple Stöber-based method wherein both the synthesis and the self-assembly of SiO_2_ nanoparticles can be facilitated by a silica-binding peptide (SiBP). We demonstrate that the SiBP acts as a multirole agent when used alone or in combination with a strong base catalyst (NH_3_). When used alone, SiBP catalyzes the hydrolysis of precursor molecules in a dose-dependent manner and produces 17–20 nm SiO_2_ particles organized in colloidal gels. When used in combination with NH_3_, the SiBP produces smaller and more uniformly distributed submicrometer particles. The SiBP also improves the long-range self-assembly of the as-grown particles into an opal-like structure by changing the surface charge, without any need for further modification or processing of the particles. The results presented here provide a biomimetic route to the single-step synthesis and assembly of SiO_2_ nanoparticles into colloidal gels or opal-like structures.

## Introduction

Ordered structures of nanoparticles have gained increased attention due to the great potential they offer in filtering, separation, drug delivery, optics, electronics, and catalysis [1–5]. Nanoparticles with ordered 3D structures, such as supra-particles or super lattices, can possess properties that are not observed in the bulk material or in individual nanoparticles [6–7]. Manufacturing such structures in a well-defined, controllable, and scalable manner is an ongoing challenge. One of the most common strategies to this end are top-down lithographic techniques. While these techniques can yield well-defined and controllable structures, high cost, labor-intensiveness, resolution limits, and high throughput time limit the scalability [8]. Self-assembly allows to circumvent some of the constraints of the top-down techniques to obtain ordered 2D or 3D nanostructures. Self-assembly, however, presents challenges of its own. One major challenge is the difficulty in manipulating nanoparticles due to size-related constraints. The self-assembly of nanoparticles is mainly governed through intermolecular interactions [9]. The high nanoparticle/volume fractions required for large-scale applications may result in electrostatic repulsion or molecular crowding-like effects, preventing efficient assembly of the particles. Therefore, tailoring intermolecular interactions between nanoparticles by modifying the particle surfaces or through external influences such as temperature, pH value, templates, and magnetic or flow fields, is important to achieve ordered nanostructures [9–14]. Although these methods can increase the efficiency of the self-assembly, they can also complicate the fabrication process further, sometimes even more than the top-down approaches. Therefore, there is still a need for simple methods to synthesize monodisperse nanoparticles and to modify the surface properties to fully exploit the advantages offered by self-assembly.

Biomolecules, such as peptides and proteins, have been demonstrated to be useful in the synthesis and self-assembly of inorganic nanostructures [15–16]. Herein, we have investigated the utility of a silica-binding peptide (SiBP) in the single-step synthesis and self-assembly of SiO_2_ nanoparticles into ordered 3D structures. The SiBP is a member of the “solid-binding peptides” family. Solid-binding peptides are designed to have strong and often specific binding affinity to solid surfaces [17–18]. Because of their interactions with solid surfaces, these peptides have been shown to be able to functionalize nanostructures, catalyze the formation of nanostructures, and modify the nucleation, growth and self-assembly processes [19–24]. For this study, we have selected a second-generation SiBP, which was developed using a bioinformatics knowledge-based all-against-all comparison of first-generation SiBPs identified via phage display [25]. The SiBP used in the study was selected because of its high affinity to SiO_2_ and the presence of nucleophilic (serine) and basic (arginine) amino acids. The hypotheses of this study were as follows: (1) The basic serine and arginine residues in the SiBP can facilitate hydrolysis of the precursor molecules and, thus, catalyze the synthesis of SiO_2_ particles. (2) The affinity of the SiBP to SiO_2_ can narrow down the size distribution of the particles through a capping agent-like effect. (3) The SiBP can increase the efficiency of the self-assembly by modifying the net surface charge of the particle.

To test these hypotheses, we have synthesized SiO_2_ particles with the Stöber method using the SiBP as the only catalyst or in combination with NH_3_. The reaction kinetics were monitored via measuring the optical density (OD) with UV–vis spectroscopy and the conversion of substrate via gas chromatography coupled with mass spectroscopy (GC–MS). Size and net surface charge distribution of the particles were determined with dynamic light scattering (DLS). The efficiency of the self-assembly was evaluated with scanning electron microscopy (SEM), UV–vis spectroscopy, and qualitative visual demonstration.

## Results and Discussion

### SiBP alone as catalyst

**Figure 1:**
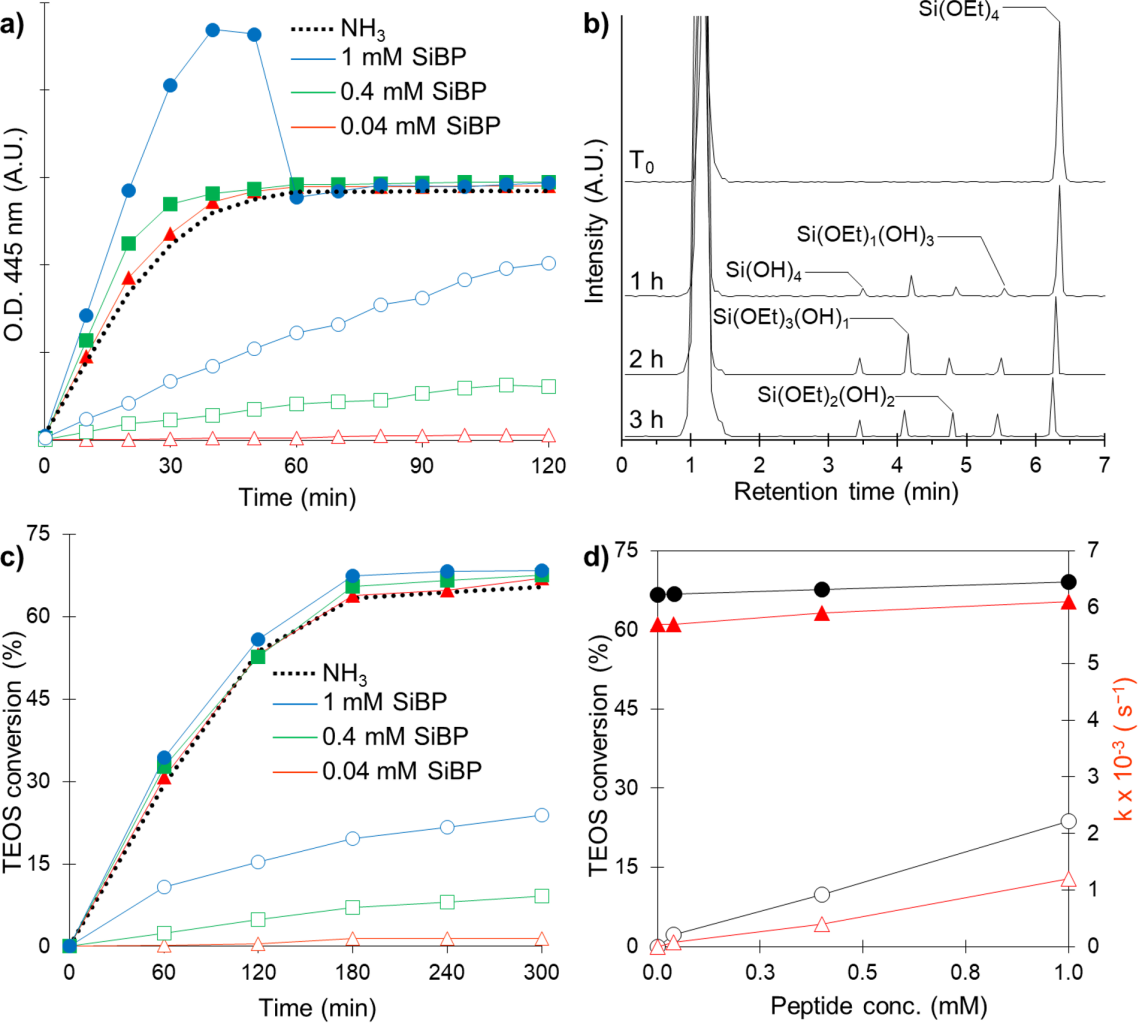
(a) OD profiles of particle formation (dashed line: no SiBP, empty markers: SiBP alone, solid markers: SiBP + 0.45 M NH_3_). (b) GC spectra of the control (NH_3_ only) group at different time points. (c) TEOS hydrolysis rates (dashed line: no SiBP, empty markers: SiBP alone, solid markers: SiBP + 0.45 M NH_3_). (d) Effect of peptide concentration on reaction rate and yield (circles: TEOS conversion at 120 min, triangles: rate constant, empty markers: SiBP alone, solid markers: SiBP + 0.45 M NH_3_).

Reaction kinetics were studied via OD measurements of the particles and GC analysis of tetraethyl orthosilicate (TEOS). OD profiles of the particle formation are shown in Figure 1a, and representative GC spectra of the control group are shown in Figure 1b. No particle formation or TEOS hydrolysis was observed in the negative control (no catalyst) group within the measurement timeframe. As seen from the OD profiles (Figure 1a) and TEOS conversion rates (Figure 1c), distinct profiles were observed when SiBP was added alone or in combination with NH_3_.

SiBP was able to hydrolyze TEOS and produce SiO_2_ particles when used alone. The increase in peptide concentration resulted in a dose-dependent increase in the reaction rate and yield (Figure 1d). Despite the same amount of precursors added to all reactions, the yields of the reactions with SiBP alone at plateau were lower compared to reactions containing NH_3_. Possible reasons of this observation will be discussed below.

**Figure 2:**
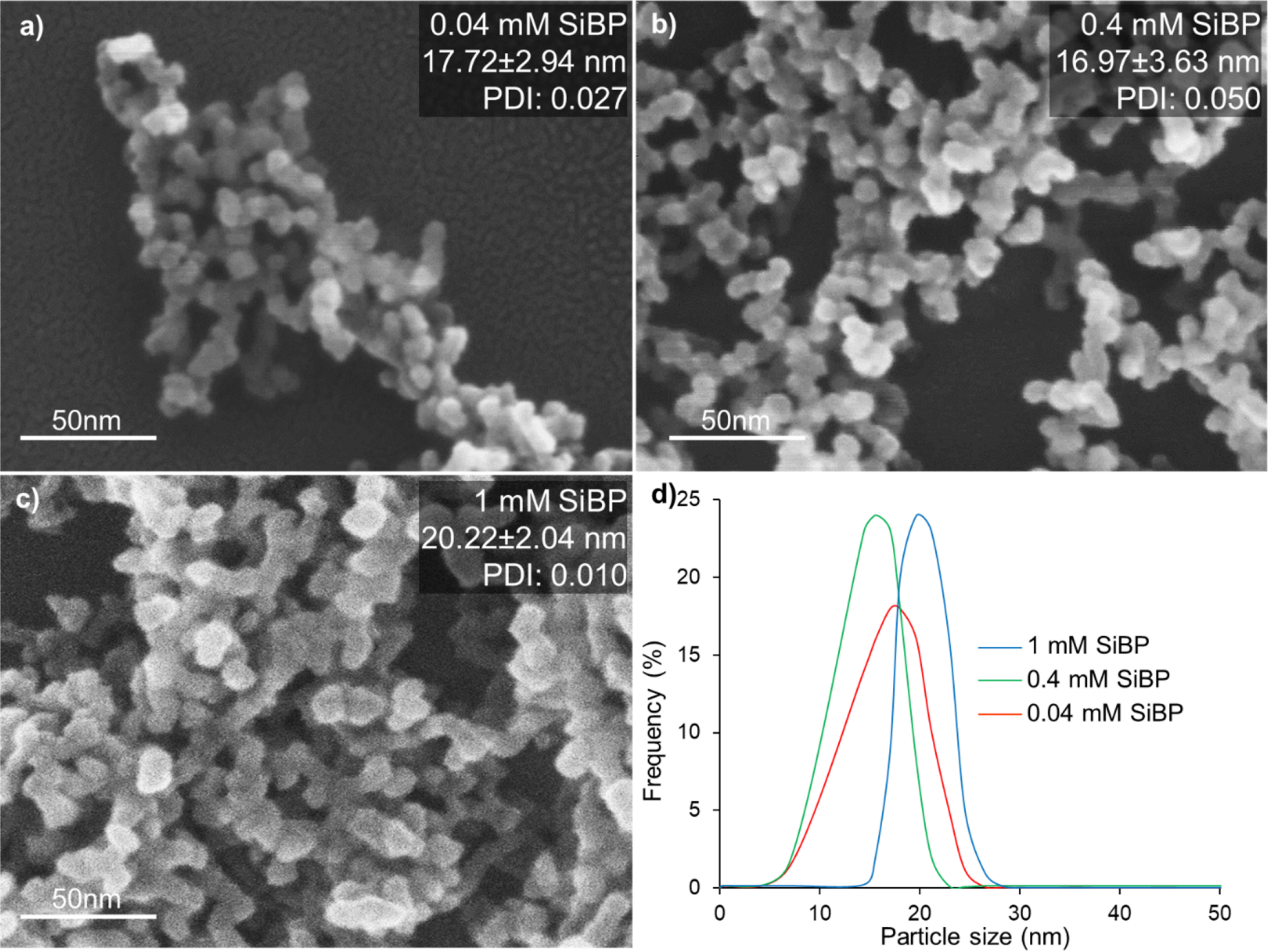
SEM images of the SiO_2_ particles formed by SiBP alone at concentrations of (a) 0.04 mM, (b) 0.4 mM, and (c) 1 mM. (d) Particle size frequency distributions obtained from DLS measurements.

No significant differences were observed regarding particle size or morphology depending on the SiBP concentration when SiBP was used alone as catalyst (Figure 2). At all concentrations, 17–20 nm particles were obtained with polydispersity indices (PDI) of 0.027, 0.050, and 0.010 for 0.04, 0.40, and 1 mM SiBP, respectively, indicating highly monodisperse (PDI < 0.080) particles. At all concentrations, the particles were organized into a branched fibrillar network, which is characteristic to the gel state of colloidal SiO_2_ (Figure 2) [26]. Colloidal gels are formed when colloidally suspended particles form a branched fibrillar network of particle strands through interparticle attractions. Under alkaline conditions, electrostatic repulsion between the SiO_2_ particles prevents formation of interconnecting particle strands. However, the presence of a cationic emulsifier allows for stable interparticle interactions and coagulation of the particles into interconnected particle strands [26]. Our findings indicate that, when used alone, the positively charged SiBP can also act as a cationic emulsifier resulting in the branched fibrillar networks observed by SEM.

In this aspect, when used alone, the SiBP mimicked the in vitro behavior of biosilicification-related proteins (BSRPs), such as silicateins and silaffins. BSRPs facilitate the formation of inorganic silica structures in marine organisms [27–28]. It has been reported that isolated silicateins and silaffins or certain repeating motifs of these proteins can facilitate silica precipitation in vitro [29–32]. The catalytic activity of these proteins is thought to be similar to the serine–histidine–aspartic acid (SHD) catalytic triad [33–34]. In this model, a hydrogen bond between serine and histidine increases the nucleophilicity of serine. Aspartic acid stabilizes the favorable orientation of histidine. Then a nucleophilic attack by serine on the Si–O bond of the precursor molecule results in a Ser–O–Si(OR)_3_ transitory complex. The hydrolysis is completed by the addition of water, separating the protein and the hydrolyzed precursor molecule, and the release of ethanol.

Although SiBP contains an N-terminal serine and two arginine residues, it does not contain histidine, aspartic acid, or another residue that can act as a H bond acceptor for serine. However, serine residues can form hydrogen bonds among themselves. Therefore, one can speculate that a hydrogen bond formed between the serine residues of two peptide molecules can increase the nucleophilicity. If this is the case, the nucleophilic attack of serine can facilitate hydrolysis of TEOS. However, a second and more likely speculation is that the SiBP mediates the hydrolysis through arginine residues. Arginine is a very strong proton acceptor with a side chain p*K*_a_ of 13.80 [35]. Therefore, locally increased concentrations of OH^−^ by arginine could facilitate the hydrolysis of silicon alkoxides, since OH^−^ is also a potent nucleophile. Future studies where serine and arginine residues of the SiBP are substituted could help to elucidate the nature of the catalytic activity of SiBP.

As mentioned above, the yield of the reactions with SiBP alone were lower compared to reactions including NH_3_ despite the same amount of initial precursor molecules (Figure 1c). A probable reason of this observation is the high affinity of the peptide to SiO_2_ [25]. As colloidally stable silica particles start to form, the peptide starts to adhere to the surface of the particles, effectively being removed from the solution before all the precursor molecules available are hydrolyzed.

### SiBP + NH_3_ as catalyst

When NH_3_ was added, the SiBP had a negligible effect on the reaction rate and the yield (Figure 1c), indicating that SiBP contributed very little to the catalytic process. This can also be the result of high affinity of SiBP to SiO_2_. Since particle formation occurs relatively fast when NH_3_ is added, it is possible that SiBP binding to the particles results in the negligible effect on reaction rate and yield.

**Figure 3:**
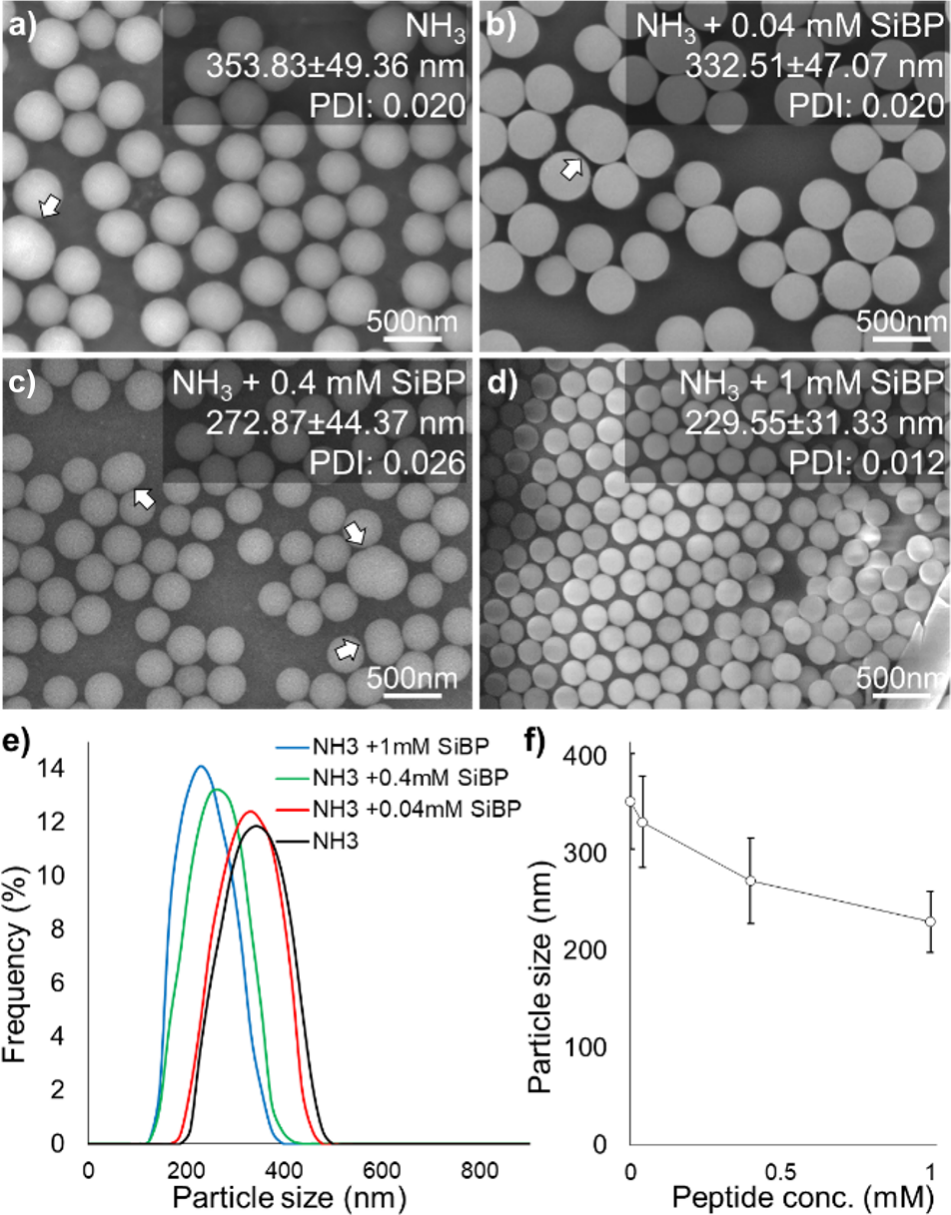
SEM images and size distributions of the particles formed with (a) NH_3_ only and NH_3_ with (b) 0.04 mM, (c) 0.4 mM, and (d) 1 mM SiBP (arrows: coalescing particles). (e) Particle size distributions obtained from DLS measurements. (f) Effect of SiBP on the particle size when NH_3_ was added.

All groups containing NH_3_ yielded spherical submicrometer particles characteristic to the Stöber method (Figure 3a–d). However, a decrease in average particle size and size distribution was observed with increasing SiBP concentrations (Figure 3e,f). The PDI for the NH_3_ + 1 mM SiBP was lower compared to other groups containing NH_3_, indicating a narrower size distribution. However, the other groups also yielded monodisperse distributions with PDIs below 0.080 (Figure 3a–d). The second hypothesis of the study is that because of its high affinity to SiO_2_, SiBP can form a dense layer on the particle surface that improves the stability of nanoparticles [36]. These observations support this hypothesis, as smaller and more narrowly distributed particles were obtained with higher SiBP concentrations. Capping agents are not only used to regulate growth and size of colloidal nanoparticles, but also to control the physicochemical or biological characteristics. As will be demonstrated below, in addition to the size of the particles, the SiBP also changes the surface charge of the particles, resulting in improved self-assembly.

**Figure 4:**
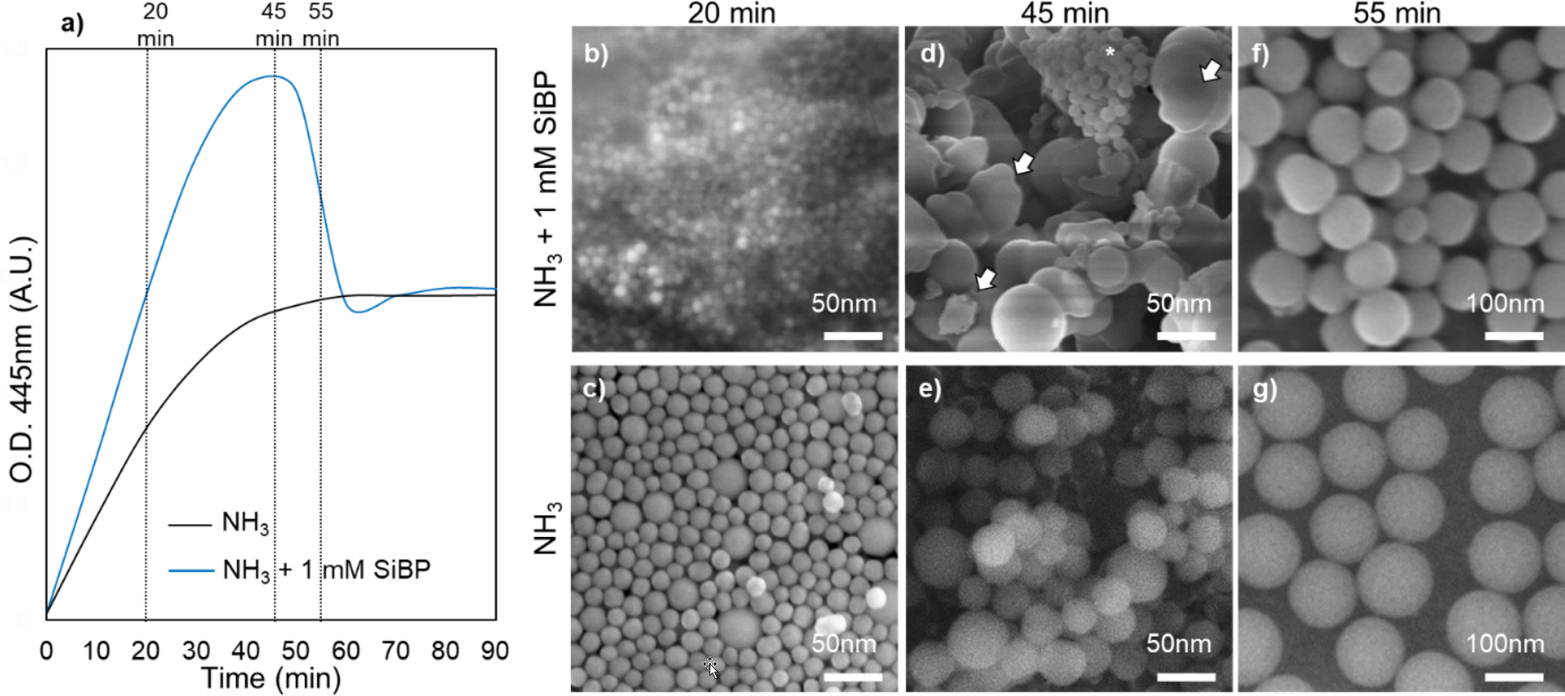
(a) UV–vis spectra of the reactions with NH_3_ alone and NH_3_ + 1 mM SiBP. SEM images of the SiO_2_ particles collected after (b, c) 20 min, (d, e) 45 min, and (f, g) 55 min (arrows: coalescing particles, asterisk: clusters of approx. 10 nm particles).

An interesting and distinct OD profile was observed in the NH_3_ + 1 mM SiBP reaction, which started with a fast increase reaching to a peak higher than the plateau of the other groups after approximately 40 min. This was followed by a steep decrease falling back to the plateau of the other groups after approximately 20 min (Figure 1a). This was at first thought to be the result of an error in experimental methods or measurement. The reactions were repeated with freshly prepared solutions and different brands of 96-well plates, but the distinct profile was observed in each repetition. To further investigate this interesting profile, samples were collected from the NH_3_ + 1 mM SiBP reaction and the NH_3_ only reaction in the middle of the steep increase (20 min), at the peak point (45 min), and in the middle of the steep decrease (55 min) (Figure 4a). SEM analysis showed that very different particle formation regimes occurred in the presence and absence of the SiBP. After 20 min, NH_3_ + 1 mM SiBP yielded a high amount of monodisperse particles of approx. 10 nm (Figure 4b), while NH_3_ alone yielded polydisperse particles of 10–40 nm (Figure 4c). After 45 min, NH_3_ + 1 mM SiBP yielded a mixture of irregularly shaped particles (Figure 4d; arrows), clusters of small 10 nm particles (Figure 4d; asterisk), and larger particles between 30 and 50 nm, while NH_3_ alone yielded spherical particles of 20 to 50 nm (Figure 4e). After 55 min, NH_3_ + 1 mM SiBP yielded particles of 60–100 nm. Small particle clusters or irregular particles were not observed (Figure 4f), while NH_3_ alone yielded spherical particles of 100 to 130 nm (Figure 4g).

Based on the OD and SEM observations, SiBP seems to drastically change the particle formation/growth regime above a threshold concentration. In the reactions of NH_3_ alone, NH_3_ + 0.04 mM SiBP, and NH_3_ + 0.4 mM SiBP, the growth regime followed the classical aggregative growth and monomer addition model [37]. According to this model, at the early stages of the reaction, the dominant regime is homogeneous nucleation of SiO_2_ particles. The growth continues by coalescence and Ostwald ripening of the particles. Growth by coalescence and Ostwald ripening is a fundamental process that plays a dominant role in nanoparticle formation. In coalescence, two or more particles combine to form a larger particle, whereas in Ostwald ripening, small particles dissolve in a solution and redeposit to form large masses. The process is mainly driven by the differences in chemical potential and surface energy between particles with different size and shape. In SiO_2_ synthesis, smaller particles with high surface energy dissolve via cleavage of siloxane bonds on the surface. The released silicic acid is then deposited onto particles with larger radius. Evidence of coalescence was observed in our study as well (shown by the arrows in Figure 3 and Figure 4). At later stages of the reaction, when the precursor concentration drops below the nucleation threshold, the dominant regime becomes growth by monomer addition to the surface of the particles.

At 1 mM concentration, however, the SiBP alters this profile and delays the aggregation of early approx. 10 nm particles into larger particles (Figure 4b). The most likely mechanism for this is the SiBP binding on the surface of the particles and, in turn, stabilizing the early particles. The resulting high number of small particles with very large surface area results in the high OD at 45 min, which is higher than the plateau of the positive control group (Figure 4a). When the fraction of the peptide to primary particles exceeds a critical value, the primary particles rapidly aggregate into larger particles, which results in the rapid drop in the OD (Figure 4a,d). Further studies are underway to take advantage of this interesting effect in synthesizing colloidally stable approx. 10 nm SiO_2_ particles at high volume fractions.

### Self-assembly of the particles

The effect of the SiBP on the self-assembly of the as-grown particles was investigated via SEM and UV–vis spectroscopy. Single-layer and multilayer assemblies were investigated by using different dilutions of the as-synthesized particles.

**Figure 5:**
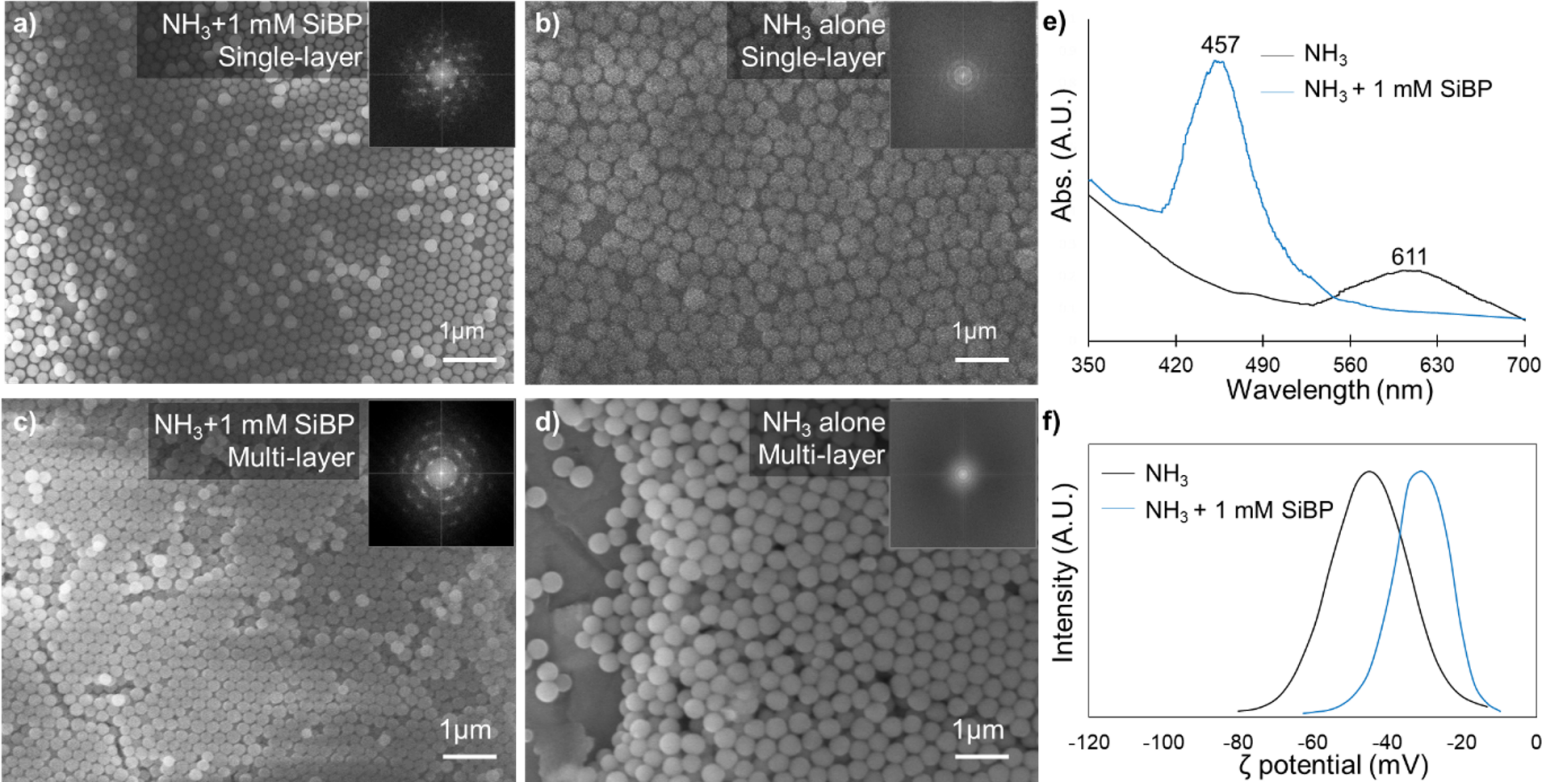
SEM micrographs of (a, c) single-layer and (b, d) multilayer self-assembled SiO_2_ particles (insets: corresponding fast Fourier transform diffraction pattern of the images). (e) UV–vis spectrogram of the self-assembled particles on a quartz surface. (f) Zeta potential of the particles in deionized water in the presence and absence of the SiBP.

SEM imaging showed that the particles from the NH_3_ + 1 mM SiBP reaction assembled into ordered single-layer (Figure 5a) or multilayer (Figure 5c) opal-like structures. Opal is a naturally occurring mineraloid with silica as the principal chemical constituent. The optical behavior of iridescent opal is a result of the regularly stratified structure of silica particles, in which the alternate layers differ in refractive index. The periodic difference in the refractive index creates photonic band gaps, in which certain wavelengths of the light cannot propagate, depending on the size of the periodic structures and the differences in the refractive indices. These structures are referred to as photonic crystals and can be manufactured synthetically for various optical applications. Similar optical behavior of the opal-like structures formed in this study will be demonstrated and discussed below.

The fast Fourier transform of the SEM images revealed a hexagonal close-packed structure (insets in Figure 5a,c). In fact, the tendency of the particles formed with NH_3_ + 1 mM SiBP to assemble into ordered structures was visible on samples not prepared by vertical deposition but simply by dripping on a surface and vacuum drying (Figure 3d). The particles formed in the reaction with NH_3_ alone assembled into less ordered single-layer (Figure 5b) or multilayer (Figure 5d) structures.

In UV–vis spectra of the self-assembled particles, a broad peak was observed around 611 nm with NH_3_ only, while with NH_3_ + 1 mM SiBP, a narrower and stronger peak was observed around 457 nm (Figure 5e). The position and the width of the absorbance peaks in the UV–vis spectroscopy are determined by the Bragg diffraction of the light in photonic crystal structures and depend on the particle size, extent of the periodicity (i.e., quality of the assembly), and the angle of the incident light. The position of the absorbance peak can be calculated using Bragg’s law (Equation 1):


[1] $$\lambda =2\sqrt{2/3}d\sqrt{fn_{{\text{SiO}}_{2}}+\left(1-f\right)n_{\text{air}}}\sin\theta ,$$

where λ is the wavelength of the peak absorbance, *d* is the diameter of the particles, *f* is the packing factor (0.74 for hcp structures), *n* is the refractive index (1.46 for SiO_2_ [38] and 1 for air), and θ is the angle of the incident light. Using this formula and the average particle sizes obtained from the particle size analysis, the absorbance peak was calculated to be 593 nm for the NH_3_-only reaction and 453 nm for the NH_3_ + 1 mM SiBP reaction. The slight difference between the calculated and measured Bragg maxima is likely due to the hydrodynamic radius measured in particle size analysis being slightly larger than the actual size of the particles. Considering this, Figure 5e shows that the calculated and measured values agree well. The broader and weaker peak observed with the NH_3_-only group is the result of the less ordered assembly and broader particle size distribution. This is better observed when the measured λ values are fitted to the theoretical Bragg reflection maximum (see Supporting Information File 1, Figure S4).

The improvement in the self-assembly by the SiBP is likely achieved by reducing the negative surface charge of the SiO_2_ particles. The surface of the SiO_2_ particles is negatively charged above pH 2–3 [39–40]. Therefore, the as-synthesized particles exhibit little cohesion because of electrostatic repulsion. Usually, a post-assembly sintering process or long waiting periods [41] are required to improve the assembly. When the positively charged SiBP binds to the particle surface, it reduces the negative charge of the particle surface (Figure 5f). The particles synthesized with NH_3_ only had a ζ potential of −44.90 mV, while the particles synthesized with NH_3_ + 1 mM SiBP had a ζ potential of −30.87 mV. At the meniscus where the solvent dries out, the decreased surface charge reduces the distance between the particles, enabling them to assemble into more ordered and close-packed structures.

**Figure 6:**
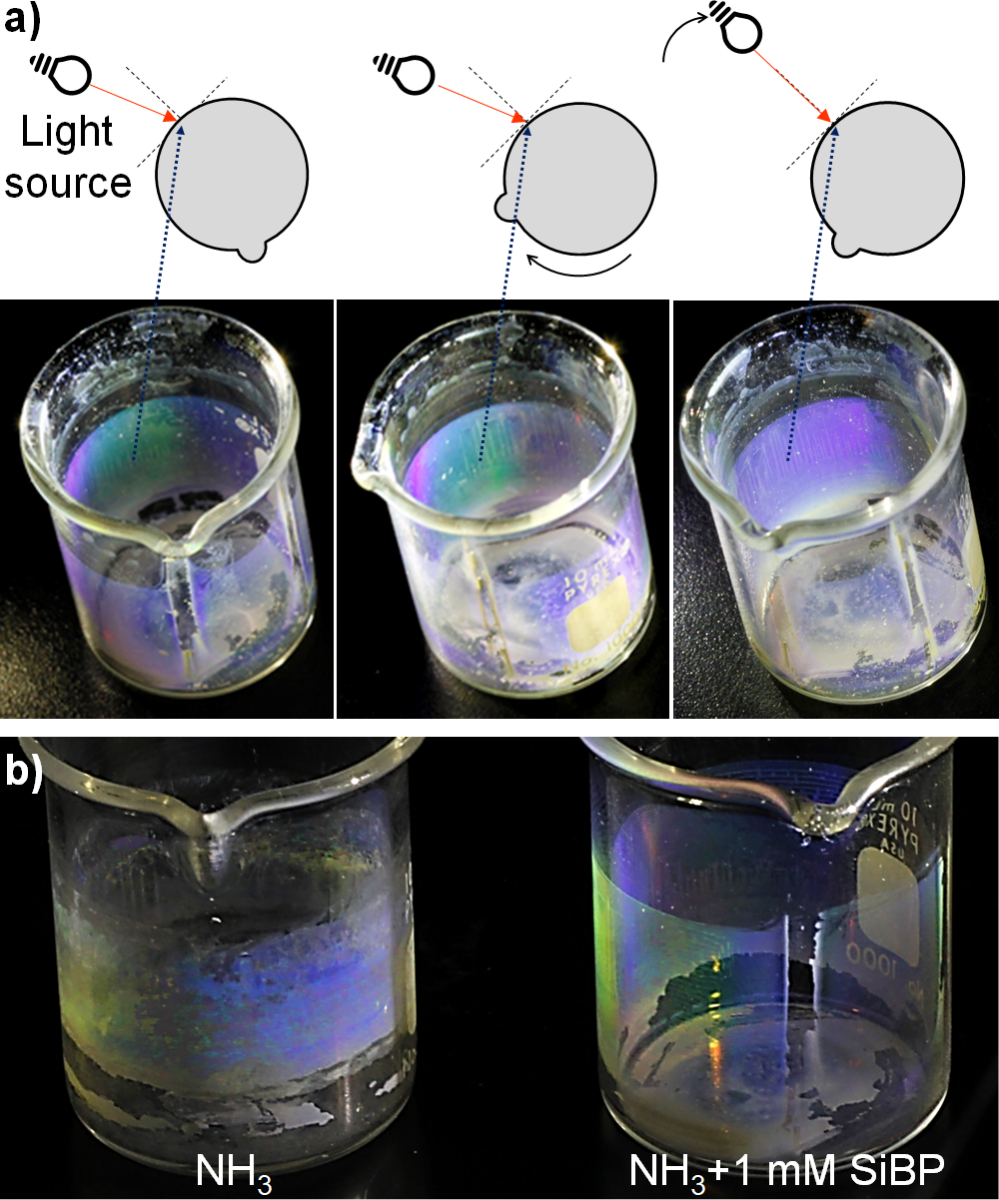
(a) Qualitative demonstration of the long-range homogeneity of self-assembly and angular dependence of Bragg reflection of as-synthesized SiO_2_ particles formed with NH_3_ + 1 mM SiBP. (b) Qualitative comparison of the long-range homogeneity of self-assembly of as-synthesized SiO_2_ particles formed with NH_3_ alone and NH_3_ + 1 mM SiBP.

Angular dependence of the Bragg reflections and the uniformity of the assembled particles from the NH_3_ + 1 mM SiBP group was qualitatively investigated by assembling the particles on the inner surface of a cylindrical beaker by letting the reaction solution evaporate under vacuum. Following the curvature of the surface, different colors were observed depending on the angle of the incident light (Figure 6a). When the beaker was rotated under the same angle of incident light, the observed colors did not change, indicating a long-range uniform assembly. Side-by-side visual comparison of the uniformity of the self-assembly from the NH_3_ alone and NH_3_ + 1 mM SiBP reactions are demonstrated in Figure 6b.

## Conclusion

We investigated the utility of a silica-binding peptide (SiBP) on SiO_2_ nanoparticle formation and self-assembly. We demonstrated that the SiBP can function in multiple ways in the synthesis and assembly processes (see Supporting Information File 1, Figure S5).

The first hypothesis of the study was accepted as when used alone, the SiBP was able to catalyze the hydrolysis of precursor molecules and precipitate approx. 20 nm SiO_2_ particles organized in a branched fiber network. We propose that the catalytic activity is mediated either by the serine residue or the arginine residues, or a combination of both.

The second hypothesis of the study was partially accepted as when used alone, the SiBP had no effect on particle size regardless of the peptide concentration. However, when used in combination with NH_3_, the SiBP resulted in smaller and more uniform particles with increased peptide concentration.

The third hypothesis of the study was accepted as the SiBP improved the self-assembly behavior by adhering on the particle surface and reducing the negative surface charge.

The results presented here provide a single-step biomimetic route to synthesis and assembly of SiO_2_ nanoparticles into gels or opal-like ordered structures.

## Experimental

### Synthesis of the peptide

The SiBP was commercially synthesized (Genscript, NJ, USA) at <98% purity via 9-fluorenylmethoxycarbonyl (F-moc) chemistry methods. The amino acid sequence and physicochemical properties of the SiBP are shown in Table 1.

**Table 1:** Amino acid sequence and physicochemical properties of the SiBP.

Amino acid sequence	Molecular weight (Da)	Isoelectric point	Grand average of hydropathy	Net charge

SPPRLLPWLRMP	1462.80	12.00	−0.317	+2

### Synthesis of SiO_2_ particles

The particles were synthesized based on the method described by Stöber and co-workers [42]. Tetraethyl orthosilicate (TEOS) (Alfa Aesar, Ward Hill, MA, USA) was used as the precursor, a 1:1 mixture of ethanol/deionized water was used as the solvent, and NH_3_ (Thermo Fischer Scientific Inc, Waltham, MA, USA) was used as the catalyst. Briefly, the Stöber process involves hydrolysis of an alkoxysilane precursor, such as TEOS, in alcohol (typically methanol or ethanol) in the presence of a catalyst. This is followed by ethanol or water condensation polymerization of the hydrolyzed precursor. Once colloidally stable nuclei form, growth ensues through monomer addition or coalescence and Ostwald ripening. The reactions involved in the Stöber process are described as follows:


[2] $${\text{NH}}_{3}+{\text{H}}_{2}\text{O}↔{\text{NH}}_{4}{}^{+}+{\text{OH}}^{-}$$

[3] $${\text{Si(OR)}}_{4}+{\text{H}}_{2}\text{O}↔{\text{(OH)Si(OR)}}_{3}+\text{ROH}$$

[4] $${\text{Si(OR)}}_{4}+{\text{(OH)Si(OR)}}_{3}↔{\text{(OR)}}_{3}{\text{Si-O-Si(OR)}}_{3}+\text{ROH}$$

[5]

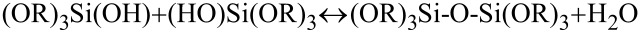



where Equation 2 is the ionization of the ammonia, Equation 3 is the hydrolysis of the alkoxysilane, Equation 4 is polymerization via alcohol condensation, and Equation 5 is polymerization via water condensation.

The reaction solvent was prepared by mixing equal volumes of gradient grade (≥99.9%) ethanol (Sigma-Aldrich, MO, USA) and deionized water (or a solution of the peptide prepared in deionized water). Then TEOS (≥99.0%) (Sigma-Aldrich, MO, USA) was added to a final concentration of 0.45 M and mixed rigorously for 10 min. The reaction solutions were transferred to a 96-well plate in triplicates for each group. The reactions were started by adding NH_3_. The reactant contents for different groups are given in Table 2. In groups 1, 2, and 3, no NH_3_ was added to investigate whether SiBP had a catalytic activity by itself to precipitate SiO_2_ particles. In groups 4, 5, and 6, 0.2 mM NH_3_ (prepared from 25% ammonium hydroxide (Merck & Co., NJ, USA)) was added in combination with different concentrations of the peptide to examine whether the peptide had a synergistic effect on the formation of SiO_2_ particles.

**Table 2:** Reaction contents for the different conditions studied.

Group	Catalyst	Precursor

blank	0.2 mM NH_3_	—
(−) control	—	0.45 M TEOS
(+) control	0.2 mM NH_3_	
group 1	0.04 mM SiBP	
group 2	0.40 mM SiBP	
group 3	1.00 mM SiBP	
group 4	0.04 mM SiBP + 0.2 mM NH_3_	
group 5	0.40 mM SiBP + 0.2 mM NH_3_	
group 6	1.00 mM SiBP + 0.2 mM NH_3_	

### Kinetic measurements

The reaction rates were monitored by measuring the optical density (OD) of the particles at 445 nm (Varioskan Flash micro-plate reader, Thermo Fischer Scientific Inc, Waltham, MA, USA) and by monitoring the hydrolysis of TEOS via gas chromatography coupled with mass spectrometry (GC–MS) (TSQ Duo Triple Quadrupole, Thermo Fischer Scientific Inc., Waltham, MA, USA). OD measurements were done at room temperature and constant mixing at 300 rpm. GC–MS analyses were done with a fused silica capillary column a (Restek 14623, Thermo Fischer Scientific Inc, Waltham, MA, USA), 150 °C injection temperature, 250 °C detection temperature, 1 µL injection volume, and 1 mL/min He flow rate. Dionex Chromeleon 7.2 software (Thermo Fischer Scientific Inc., Waltham, MA, USA) was used for the quantitative analysis of GC–MS data. The reaction rate of TEOS hydrolysis was calculated according to first-order reaction kinetics [43].

### Particle size and zeta potential measurements

Measurements were performed using a Zetasizer Nano ZS90 (Malvern Panalytical Ltd, Malvern, UK). As-synthesized particles were diluted to 1:10 in the reaction solvent (1:1 ethanol/deionized water) and dispersed by sonication for 10 min. Readings were taken at room temperature. Malvern DTS software v.5.10 (Malvern Panalytical Ltd, Malvern, UK) was used for data processing and analysis. Particle size distributions are reported based on intensity weighted averages and expressed as mean diameter ± SD. Each reading was repeated three times and readings were done with three runs (3 min/run).

### Self-assembly of SiO_2_ particles

Colloidal particles were synthesized in the same way as described in Table 2 with a final volume of 10 mL. The particles were assembled by a vertical deposition method. For SEM analysis, the particles were assembled on regular microscope cover slides. For UV–vis spectroscopy analysis, the particles were assembled on quartz slides. One end of the slide was attached to a NE-1002X micro-fluidics automatic syringe pump (New Era Pump Systems Inc., Farmingdale, NY, USA) and the other end was vertically dipped into the as-synthesized colloidal solution of particles (see Supporting Information File 1, Figure S3) The syringe pump was run at a speed of 1.50 µm/s until the covers were moved completely out of the colloidal solution.

### Scanning electron microscopy

To analyze as-synthesized particles, 50 µL aliquots of the reaction solutions were placed on standard microscope cover slides. The excess liquid was removed by absorbing on a clean absorbent paper. To analyze self-assembled particles, cover slides described in the previous section were used. The slides were dried under vacuum and then adhered onto an aluminum sample holder using carbon tape. The samples were coated with platinum for 30 s using an EM ACE200 vacuum coater (Leica Microsystems GmbH, Wetzlar, Germany). All SEM analyses were performed using a SU5000 SEM (Hitachi, Japan) at 10 kV accelerating voltage.

### UV–vis absorbance spectroscopy

The particles were assembled on quartz slides by vertical deposition as described before. UV–vis absorbance analysis was made using a T80+ UV–vis spectrophotometer (PG Instruments Ltd., Leicestershire, UK).

## Supporting Information

Supporting Information features mass spectra of the reaction with NH_3_ only at different time points, mean OD values collected from three separate syntheses, a schematic representation of the vertical deposition method used in the study, the fit of measured λ values to the theoretical Bragg reflection maxima, and a schematic representation of the multiple roles of the SiBP.

File 1 Additional experimental data.
