# Supplementary material for: Biocatalytic synthesis and ordered self-assembly of silica nanoparticles via a silica-binding peptide
Source: Beilstein J Nanotechnol. 2023 Feb 28;14:280–90. doi: 10.3762/bjnano.14.25 (PMC9989680; doi:10.3762/bjnano.14.25)
Supplement: File 1 — Additional experimental data. [file Beilstein_J_Nanotechnol-14-280-s001.pdf]

BEILSTEIN JOURNAL OF NANOTECHNOLOGY

Supporting Information

for

Biocatalytic synthesis and ordered self-assembly of silica nanoparticles via a silica-binding peptide

Mustafa Gungormus

Beilstein J. Nanotechnol. **2023,** *14,* 280–290. doi:10.3762/bjnano.14.25

Additional experimental data

License and Terms: This is a supporting information file under the terms of the Creative Commons Attribution License (https://creativecommons.org/licenses/by/4.0). Please note that the reuse, redistribution and reproduction in particular requires that the author(s) and source are credited and that individual graphics may be subject to special legal provisions.
The license is subject to the *Beilstein Journal of Nanotechnology* terms and conditions: (https://www.beilstein-journals.org/bjnano/terms)

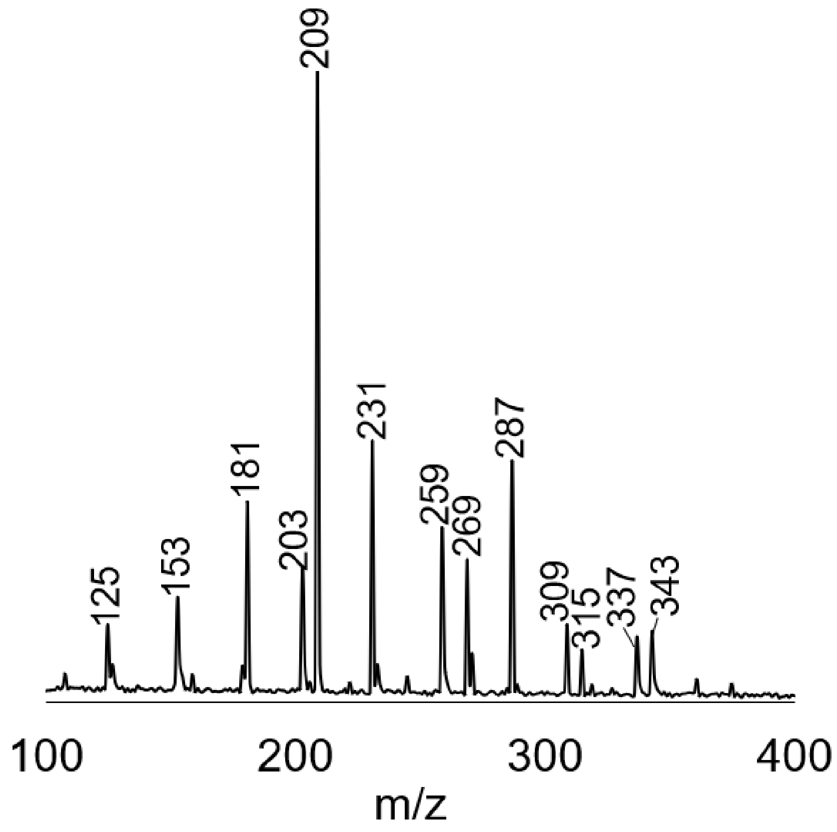

Figure S1: Mass spectra of the reaction solution of NH_3 only after 60 min.

m*/*z	**Species**
125	$[Si(OEt)_3(OH)_1]^+$
153	$[Si(OEt)_2(OH)_2]^+$
181	$[Si(OC_2H_5)_1(OH)_3]^+$
203	$[Si_2O(OEt)_1(OH)_5]^+$
209	$[Si(OEt)_4]^+$
231	$[Si_2O(OEt)_2(OH)_4]^+$
259	$[Si_2O(OEt)_3(OH)_3]^+$
287	$[Si_2O(OEt)_4(OH)_2]^+$
309	$[Si_3O_2(OC_2H_5)_2(OH)_6]^+$
315	$[Si_2O(OC_2H_5)_5(OH)_1]^+$
337	$[Si_3O_2(OC_2H_5)_3(OH)_5]^+$
343	$[Si_2O(OEt)_6]^+$

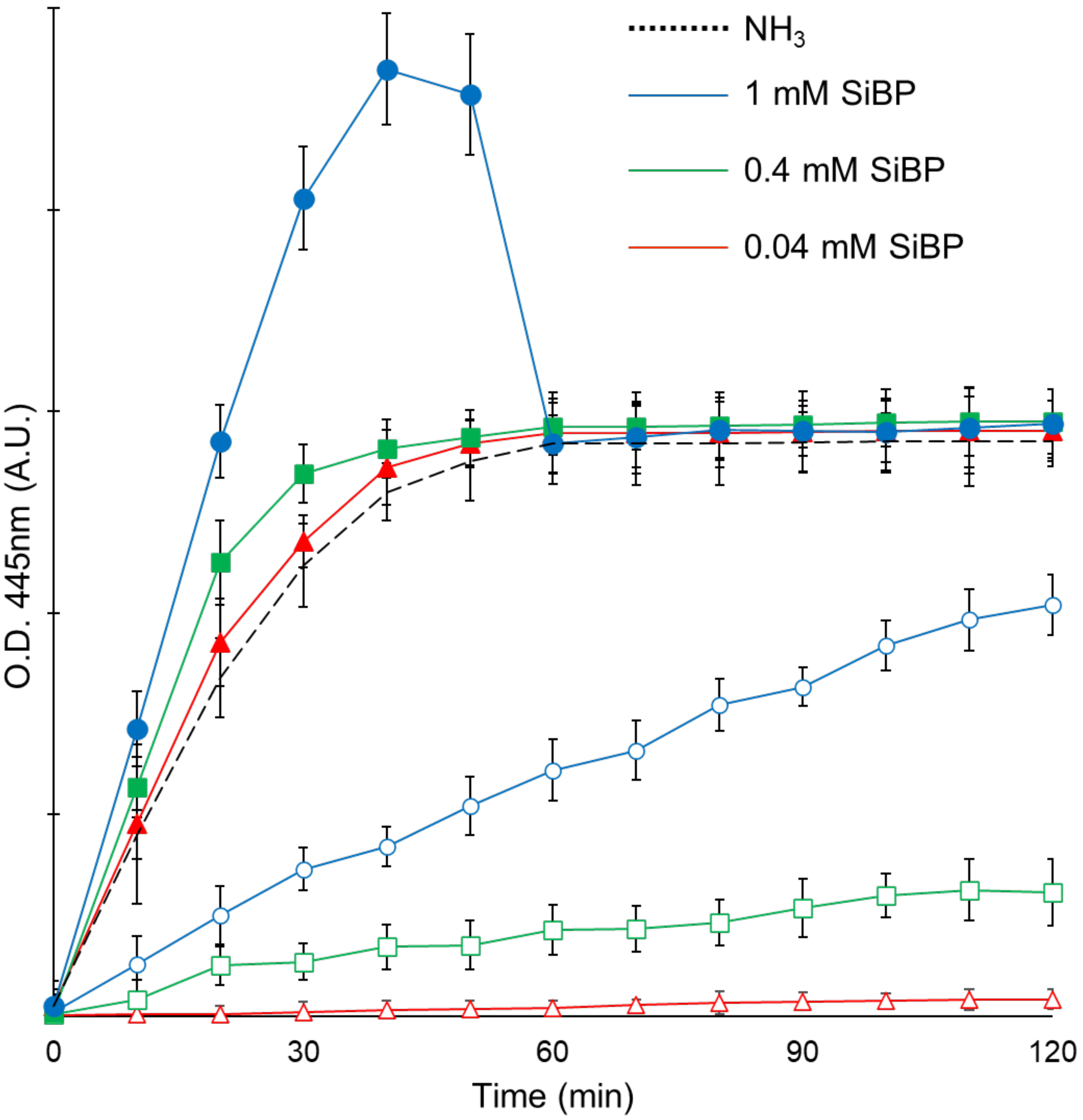

Figure S2: Mean OD value collected from three separate syntheses. (dashed line: no SiBP, empty markers: SiBP alone, solid markers: SiBP + 0.45 M NH_3).

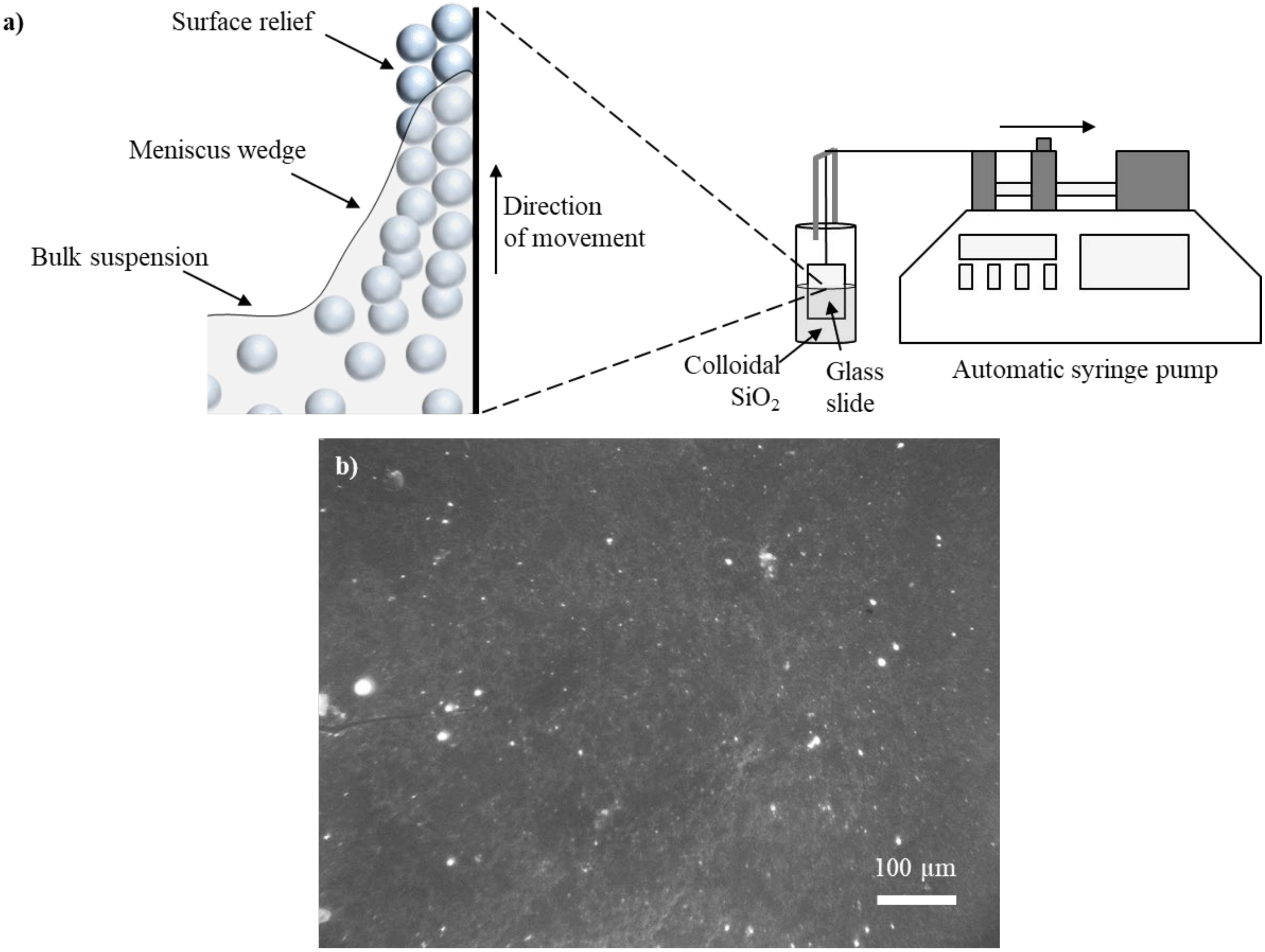

Figure S3: (a) Schematic representation of the vertical deposition method used in the study. (b) Gross assembly of SiO_2 particles on the surface.

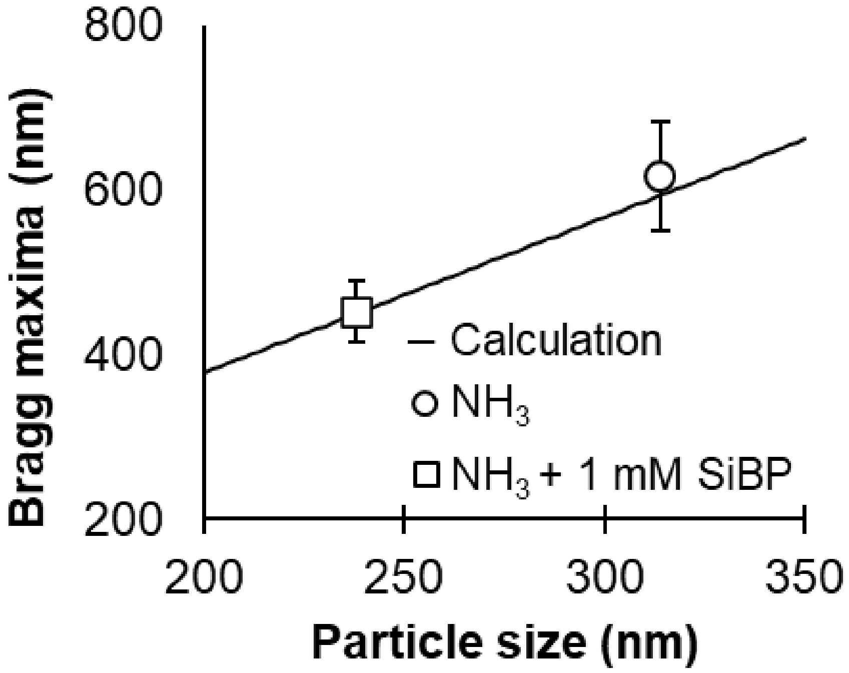

Figure S4: Fit of the measured λ values to the theoretical Bragg reflection maxima.

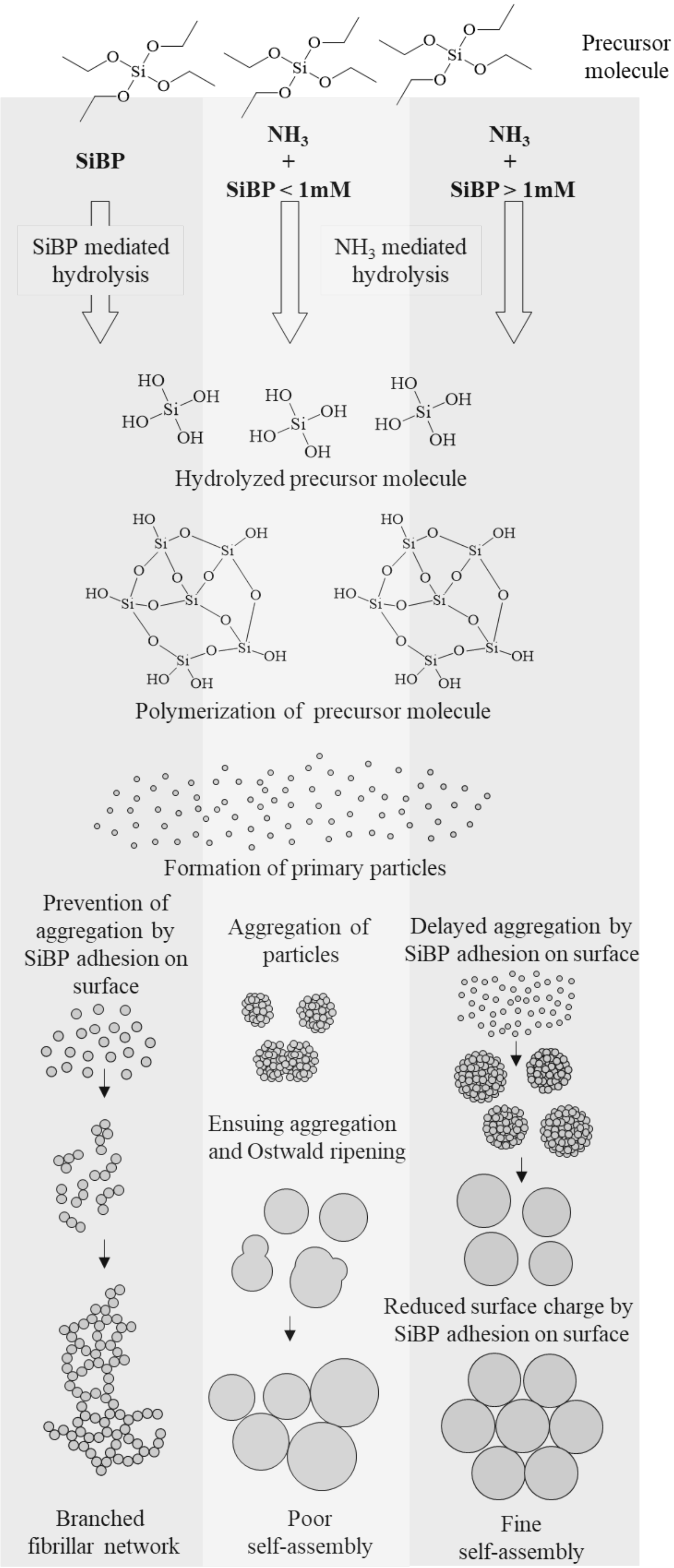

Figure S5: Schematic representation of the multiple roles of the SiBP in the formation and assembly of SiO_2.
